# Supplementary material for: Lanthanides-Based Nanoparticles Conjugated with Rose Bengal for FRET-Mediated X-Ray-Induced PDT
Source: Pharmaceuticals (Basel). 2025 May 1;18(5):672. doi: 10.3390/ph18050672 (PMC12114895; doi:10.3390/ph18050672)
Supplement: Supplementary file 1 [file pharmaceuticals-18-00672-s001.zip › pharmaceuticals-3580275-supplementary.pdf]

Supporting informations

1. Synthesis

1.1 Synthesis of NHS-activated ester derivatives of RB

The synthesis of activated **RB-NHS**, **RB-HA-NHS** and **RB-Ahx-NHS esters** is depicted in Scheme 1.

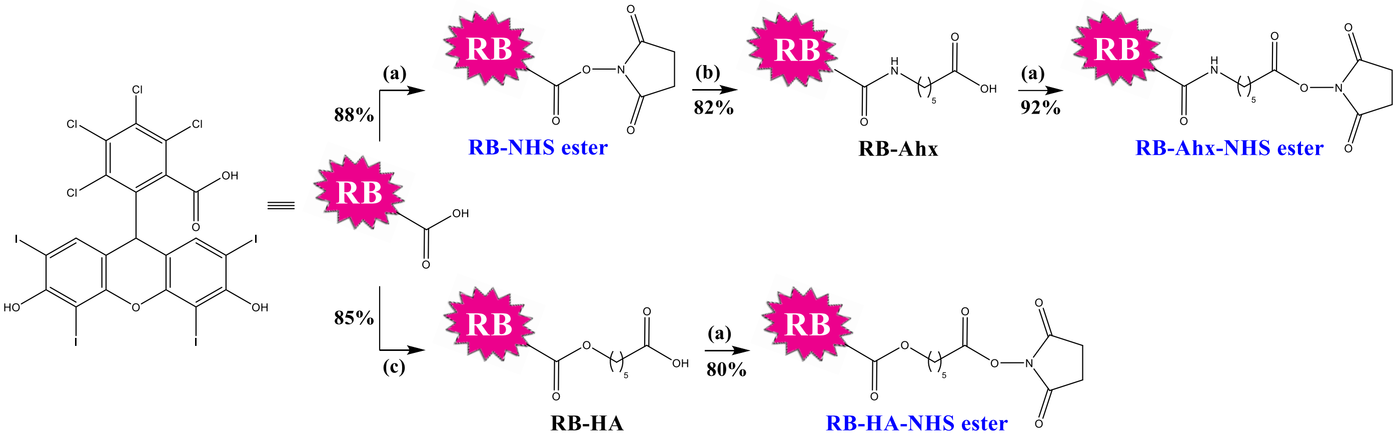

*Scheme 1: Synthesis of NHS-activated ester derivatives **RB-NHS**, **RB-HA-NHS** and **RB-Ahx-NHS**. Reagents and conditions: (a) NHS (4 eq), EDC.HCl (3 eq), DMF, RT, overnight; (b)Et_3N (1 eq), 6-aminohexanoic acid (2.5 eq), DMF, RT, 24 h; (c) 6-bromohexanoic acid (3 eq), DMF, 80°C, 24 h.*

1.1.1 Synthesis of RB-NHS ester and caracteristaion

i. Synthesis

To a solution of RB (600 mg, 0.60 mmol, 1 eq) in anhydrous DMF (50 mL) was added *N*-hydroxysuccinimide (NHS, 276 mg, 2.40 mmol, 4 eq) and *N*,*N'*-dicyclohexylcarbodiimide hydrochloride (EDC.HCl, 279 mg, 1.80 mmol, 3 eq.). The resulted mixture was stirred in the dark at room temperature under inert atmosphere overnight (Scheme 1). The solvent was then removed under *vacuum* to afford the desired **RB–NHS ester** as dark purple solid with a purity of 90% (LC-MS analysis). A mixture solvent of $CHCl_3/H_20$ (70 mL, 5/2, v/v) was used three times for extraction and the combined organic phases were dried over $MgSO_4$ and evaporated *in vacuo*. The product was purified by flash chromatography on silica gel (DCM/EtOH, 90/10, v/v).

ii. Caracterisation

ii.1. HPLC, LC-MS

RB–NHS ester (580 mg, 88%) was obtained as a dark violet solid with a 98% purity (HPLC). The purity was analysed by HPLC at a UV-Visible absorption detection wavelength (λ = 560 nm), with a gradient going from 95/5 to 0/100 (A/B, v/v) for 15 min, followed by of isocratic acetonitrile (ACN) 0/100 (A/B, v/v) for 10 min, then a gradient from 0/100 to 95/5 (A/B, v/v) in 5 min (with A = 95/5 /0.1 (H_2O/ACN/MeOH, v/v/v) and B = 100/0.1 (ACN/MeOH, v/v)).

ii.2. NMR

^1H NMR (400 MHz, DMSO-d_6): δ 2.8 (t, J = 5.9 Hz, 4H, 2 CH_2, NHS), 6.0 (br s, 2H, 2 OH), 7.5 (br s, 3H, 2 H_{arom} and 1 H_{pyr}). LC-MS (ESI) for $C_{24}H_9Cl_4I_4NO_7$ $[M+H]^+$: calcd 1071.53, found 1071; $[M+3H]^{3+}$: calcd 357.85, found 357.

1.1.2 Synthesis of RB-HA-NHS ester and caracterisation

i. synthesis

Firstly, **RB-HA** was synthesized following the slightly modified method of Necker [46]

(Scheme 1). To a solution of RB sodium salt (500 mg, 0.50 mmol, 1 eq) in DMF (20 mL) was added 6-bromohexanoic acid (HA, 284 mg, 1.5 mmol, 3 eq), and the resulting mixture was stirred at 80°C for 24 h in the dark. The solution was then allowed to cool to room temperature and acidified with H_2SO_4 (5% aqueous solution, v/v) and extracted with $CHCl_3$ (3 x 20 mL). The combined organic phases were dried over $MgSO_4$ and evaporated *in vacuo*. The resulting red oil was precipitated in cooled diethyl ether to afford the desired **RB-HA** (420 mg, 85%) as a red solid.

ii. Caracteriation

ii.1. HPLC, LC-MS

LC-MS (ESI) for $C_{26}H_{16}Cl_4I_4O_7$ $[M+H]^+$: calcd 1088.59, found 1088; $[M+Na]^+$: calcd 110.57, found 1110. **RB-HA** was used in the next step without further purification.

Secondly, to a stirred solution of **RB-HA** (400 mg, 0.35 mmol, 1 eq) in anhydrous DMF (3 mL) in the dark under inert atmosphere was added a solution of EDC.HCl (164 mg, 1.06 mmol, 3 eq) and NHS (161 mg, 1.4 mmol, 4 eq) in anhydrous DMF (2 mL). The resulted mixture was stirred in the dark at room temperature under inert atmosphere overnight (Scheme 1). The solvent was then removed under *vacuum* and a mixture solvent of $CHCl_3/H_20$ (70 mL, 5/2, v/v) was used three times for extraction. The combined organic phases were dried over $MgSO_4$ and evaporated *in vacuo*. The crude product was precipitated in cooled diethyl ether and purified by preparative HPLC using an ACN/H_2O gradient (0.1% TFA; 5/95, v/v) to 100% ACN in 20 min, followed by isocratic ACN for 15 min on a C18 Varian column (R_t =18.4 min). After lyophilization, pure **RB-HA-NHS ester** (320 mg, 80%) was afforded as a red solid.

ii.2. NMR

^1H NMR (400 MHz, DMSO-d_6): δ 1.3 (m, 2H, CH_2, HA spacer arm), 1.6 (m, 2H, CH_2, HA spacer arm), 2.2 (m, 2H, CH_2, HA spacer arm), 2.8 (t, J = 4.5 Hz, 4H, 2 CH_2, NHS), 2.9 (t, J = 7.2 Hz, 2H, CH_2CO, HA spacer arm), 3.8 (t, J = 7.2 Hz, 2H, CH_2O, HA spacer arm), 6.0 (br s, 2H, 2 OH), 7.6 (br s, 3H, 2 H_{arom} and 1 H_{pyr}). LC-MS (ESI) for $C_{30}H_{19}Cl_4I_4NO_9$ $[M+H]^+$: calcd 1185.60, found 1186.

1.1.3 Synthesis of RB-Ahx-NHS ester and caracterisation

i. Synthesis

Firstly, **RB-Ahx** was synthesized using **RB-NHS ester** (Scheme 1). To a solution of **RB-NHS ester** (100 mg, 0.093 mmol, 1 eq) in anhydrous DMF (10 mL) was added Et_3N (13 µL, , 0.093 mmol, 1 eq), then a solution of 6-aminohexanoic acid (Ahx, 83 mg, 0.23 mmol, 2.5 eq) in anhydrous DMF (4 mL). The resulting mixture was stirred at room temperature for 24 h in the dark. The solvent was then removed under *vacuum* and a mixture solvent of $CHCl_3/H_20$ (70 mL, 5/2, v/v) was used three times for extraction. The combined organic phases were dried over $MgSO_4$ and evaporated *in vacuo*.

ii. Caracteriation

ii.1. HPLC, LC-MS

RB-Ahx (82 mg, 82%) was obtained as a dark red solid with a 96% purity (HPLC). The purity was analysed by HPLC with the same conditions as described for **RB–NHS ester**. LC-MS (ESI) for $C_{26}H_{17}Cl_4I_4NO_6$ $[M+H]^+$: calcd 1087.60, found 1087. **RB-Ahx** was used in the next step without further purification.

Secondly, to a stirred solution of **RB-Ahx** (82 mg, 0.075 mmol, 1 eq) in anhydrous DMF (2 mL) in the dark under inert atmosphere was added a solution of EDC.HCl (1.05 mg, 0.225 mmol, 3 eq) and NHS (1.035 mg, 0.3 mmol, 4 eq) in anhydrous DMF (1.5 mL). The resulted mixture was stirred in the dark at room temperature under inert atmosphere overnight

(Scheme 1). The solvent was then removed under *vacuum* and a mixture solvent of $CHCl_3/H_20$ (70 mL, 5/2, v/v) was used three times for extraction. The combined organic phases were dried over $MgSO_4$ and evaporated *in vacuo*. The product was purified by flash chromatography on silica gel (DCM/EtOH, 90/10, v/v). **RB–Ahx-NHS ester** (75 mg, 92%) was obtained as a dark red solid with a 98% purity (HPLC). The purity was analysed by HPLC with the same conditions as described for **RB–NHS ester**.

ii.2. NMR

^1H NMR (400 MHz, DMSO-d_6): δ 1.2 (m, 2H, CH_2, Ahx spacer arm), 1.5 (m, 2H, CH_2, Ahx spacer arm), 2.0 (m, 2H, CH_2, Ahx spacer arm), 2.5 (m, 2H, CH_2CO, Ahx spacer arm), 2.8 (t, J = 5.0 Hz, 4H, 2 CH_2, NHS), 3.1 (t, J = 7.4 Hz, 2H, CH_2NH, Ahx spacer arm), 5.8 (br s, 2H, 2 OH), 7.2 (br s, 3H, 2 H_{arom} and 1 H_{pyr}), 7.3 (m, 1H, NH, Ahx spacer arm). LC-MS (ESI) for $C_{30}H_{20}Cl_4I_4N_2O_8$ $[M+H]^+$: calcd 1184.62, found 1184; $[M\text{-}NHS+2Na]^+$: calcd 1131.57, found 1131.

1.2 Synthesis of Fmoc-K(RB), Mal-K(RB) and Mal-K(RB)DKPPR and caracteristaion

The synthesis of **Fmoc-K(RB)** (*i.e.*, Fmoc-K(RB)-OH), **Mal-K(RB)** (*i.e.*, Mal-K(RB)-OH) and **Mal-K(RB)DKPPR** (*i.e.*, Mal-K(RB)DKPPR-OH) is depicted in Scheme 2 [45].

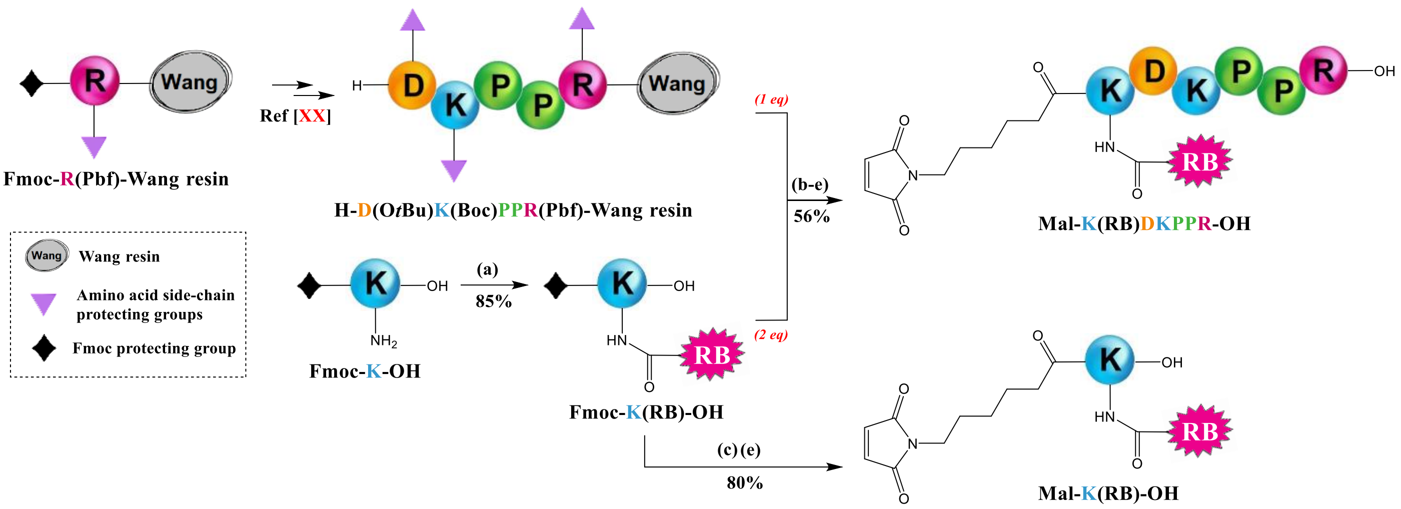

*Scheme 2: Synthesis of **Fmoc-K(RB)** (i.e., Fmoc-K(RB)-OH), **Mal-K(RB)** (i.e., Mal-K(RB)-OH) and **Mal-K(RB)DKPPR** (i.e., Mal-K(RB)DKPPR-OH). Reagents and conditions: (a) **RB-NHS** (1 eq), Et_3N (2 eq), DMF, RT, 24 h; (b) HBTU (5 eq), NMP (3 eq), NMM (10 eq), DMF, RT, 4 days; (c) Piperidine/DMF (20/80, v/v), RT, 3 x 15 min; (d) TFA/TIPS/H_20 (92.5/5/2.5, v/v/v), RT, 2 h; (e) 6-maleimidohexanoic acid (3 eq), HBTU (3 eq), NMP (3 eq), NMM (9 eq), DMF, RT, 2 days.*

1.2.1. Synthesis of Fmoc-K(RB) and caracterisation

i. Synthesis

To a stirred solution of **RB-NHS ester** (580 mg, 0.54 mmol, 1 eq) in anhydrous DMF (30 mL) in the dark under inert atmosphere was added a solution of Fmoc-L-Lys-OH.HCl (222 mg, 0.54 mmol, 1 eq) in anhydrous DMF (3 mL) and Et_3N (168 µL, 1.08 mmol, 2 eq). The resulted mixture was stirred in the dark at room temperature under inert atmosphere during 24 h (Scheme 2). The solvent was then removed under *vacuum* and the product was purified by flash chromatography on silica gel (DCM/EtOH, 90/10, v/v). **Fmoc-K(RB)** (610 mg, 85%) was obtained as a red solid with a 98% purity (HPLC).

ii. Caracterisation

ii.1. HPLC, LC-MS

The purity was analysed by HPLC with the same conditions as described for **RB–NHS ester**.

ii.2. NMR

^{1}H NMR (400 MHz, DMSO-d_6): δ 1.3 (m, 2H, γ-CH_2, Lys), 1.5 (m, 4H, β-CH_2 and δ-CH_2, Lys), 3.1 (m, 2H, ε-CH_2, Lys), 3.9 (m, 2H, α-CH, Lys, and CH, Fmoc), 4.5 (m, 2H, CH_2, Fmoc), 7.1-7.4 (m, 8H, 8 H_{arom}, Fmoc), 7.3 (m, 1H, ε-NH, Lys), 7.5 (s, 3H, 2 H_{arom} and 1 H_{pyr}, RB), 7.8 (m, 1H, α-NH, Lys). LC-MS (ESI) for $C_{41}H_{28}Cl_4I_4N_2O_8$ $[M+H]^+$: calcd 1324.68, found 1325; $[M+2H]^{2+}$: calcd 662.85, found 663.

1.2.2. Synthesis of Mal-K(RB) and caracterisation

i. Synthesis

The *N*-terminal Fmoc deprotection of **Fmoc-K(RB)** (120 mg, 0.093 mmol, 1eq) was performed using a solution of 20% piperidine in DMF (10 mL) at room temperature during 4 h in the dark. The solvent was then removed under *vacuum* and a mixture solvent of $CHCl_3/H_20$ (10 mL, 2/1, v/v) was used for extraction. The organic phase was dried over $MgSO_4$ and evaporated *in vacuo*. The resulting crude deprotected **H-K(RB)** was coupled to 6-maleimidohexanoic acid (3 eq) using hexafluorophosphate benzotriazole tetramethyl uronium (HBTU, 3 eq), *N*-methyl-2-pyrrolidone (NMP, 3 eq) and *N*-methylmorpholine (NMM, 9 eq) in DMF. The reaction was stirred at room temperature in the dark under inert atmosphere for 2 days.

ii. Caracterisation

ii.1. HPLC, LC-MS

The solvent was then removed under *vacuum* and the desired crude **Mal-K(RB)** was purified by preparative HPLC on a C18 Varian column (R_t =14.9 min). using a gradient going from 90/10 to 0/100 (A/B, v/v) for 15 min, followed by of isocratic ACN 0/100 (A/B, v/v) for 10 min, then a gradient from 0/100 to 90/10(A/B, v/v) in 5 min (with A = 100/0.1 (H_2O/TFA, v/v) and B = 100/0.1 (ACN/TFA, v/v)). After lyophilization, pure **Mal-K(RB)** (80 mg, 80%) was afforded as a pink powder with a 95% purity (HPLC). The purity was analysed by HPLC with the same conditions as described for **RB–NHS ester**.

ii.2. NMR

^{1}H NMR (400 MHz, DMSO-d_6): δ 1.2 (m, 2H CH_2, Mal), 1.3 (m, 2H, γ-CH_2, Lys), 1.6 (m, 6H, δ-CH_2, Lys, 2 CH_2, Mal), 1.8 (m, 2H, β-CH_2, Lys), 2.9 (m, 2H, CH_2CO, Mal), 3.1 (m, 1H, ε-CH_2, Lys), 3.4 (CH_2N, Mal), 4.3 (m, 1H, α-CH, Lys), 7.3 (m, 1H, ε-NH, Lys), 7.5 (s, 3H, 2 H_{arom} and 1 H_{pyr}, RB), 7.8 (m, 1H, α-NH, Lys). LC-MS (ESI) for $C_{36}H_{29}Cl_4I_4N_3O_9$ $[M+H]^+$: calcd 1295.69, found 1295.

1.2.3. Synthesis of Mal-K(RB)DKPPR and caracterisation

i. Synthesis

The peptide grafted on Wang resin **H-D(O*t*Bu)K(Boc)PPR(Pbf)-Wang resin** was synthesized as previously described by our team [45] using the ResPepXL automated peptide synthesizer, with Fmoc/tBu methodology (Scheme 2).

Following the synthesis of **H-D(O*t*Bu)K(Boc)PPR(Pbf)-Wang resin**, the coupling reaction with **Fmoc-K(RB** (2 eq) was carried out using HBTU (5 eq), NMP (3 eq) and NMM (10 eq) in DMF, and the reaction mixture was stirred for 4 days. The *N*-terminal Fmoc deprotection was performed using 20% piperidine in DMF at room temperature during 15 min (three times) to afford the resulting peptide on Wang resin, **H-K(RB)-D(O*t*Bu)K(Boc)PPR(Pbf)-Wang resin**.

Finally, **H-K(RB)-D(O*t*Bu)K(Boc)PPR(Pbf)-Wang resin** was coupled to 6-maleimidohexanoic acid (3 eq) using HBTU (3 eq), NMP (3 eq) and NMM (9 eq) in DMF. The reaction was stirred at room temperature in the dark for 2 days. The obtained resin was dried under *vacuum* and then cleaved (with full deprotection of lateral chains) using a mixture of trifluoroacetic acid/triisopropylsilane/ water (TFA/TIPS/H_20, 92.5/2.5/5, v/v/v) for 2 h. The resin was filtered and washed with TFA (2 mL) and DCM (50 mL). The filtrate was dried *in vaccuo* and freeze-dried.

ii. Caracterisation

ii.1. HPLC, LC-MS

The crude lyophilized **Mal-K(RB)DKPPR** peptide was purified by preparative HPLC on a C18 Varian column (R_t =11.2 min). using a gradient going from 90/10 to 0/100 (A/B, v/v) for 15 min, followed by of isocratic ACN 0/100 (A/B, v/v) for 10 min, then a gradient from 0/100 to 90/10(A/B, v/v) in 5 min (with A = 100/0.1 (H_2O/TFA, v/v) and B = 100/0.1 (ACN/TFA, v/v)). After lyophilization, pure **Mal-K(RB)DKPPR)** (50 mg, 56%) was afforded as a pink powder with a 92% purity (HPLC). The purity was analysed by HPLC with the same conditions as described for **RB–NHS ester**.

ii.2. NMR

^{1}H NMR (400 MHz, DMSO-*d*$_6$) chemical shifts are given in Table 4. LC-MS (ESI) for $C_{62}H_{72}Cl_4I_4N_{12}O_{16}$ $[M+H]^+$: calcd 1889.02, found 1189; $[M+2H]^{2+}$: calcd 945.01, found 945.

*Table S1: ^{1}H NMR chemical shifts of **Mal-K(RB)DKPPR** (400 MHz, DMSO–d$_6$)*

	NH	αH	βH	δH	Others
**RB**	-	-	-	-	7.8 (br s, 3H, 2 H_{arom} and 1 H_{pyr})
**Arm- Maleimide**	-	-	-	-	**Maleimide**: 6.80 (s, 2H, CH=CH) **Arm**: 3.10 (m, 2H, CH_2N), 1.37 (m, 4H, 2 CH_2) 1.09 (m, 2H, CH_2), 2.08 (m, 2H, CH_2CO)
**K**	8.30	4.00	1.78	1.70	γ-CH_2 = 1.45, δ-CH_2 = 1.70, ε-CH_2 = 3.20, ε-NH_3^+ = 7.40
**D**	8.30	4.45	2.60, 2.80	-	-
**K**	8.30	4.00	1.78	1.70	γ-CH_2 = 1.45, δ-CH_2 = 1.70, ε-CH_2 = 3.20, ε-NH_3^+ = 7.40
**P**	-	4.23	2.00, 2.10	3.21, 3.32	γ-CH_2 = 1.70
**P**	-	4.23	2.00, 2.10	3.21, 3.32	γ-CH_2 = 1.70
**R**	8.30	4.40	1.80, 1.90	3.30	γ-CH_2 = 1.70, ε-NH = 7.10

1.3 Synthesis of AGuIX Ln@RB, AGuIX Ln@spacer arm-RB, AGuIX Ln@Mal-K(RB) and AGuIX Ln@Mal-K(RB)DKPPR

The synthesis of **AGuIX Ln@RB**, **AGuIX Ln@spacer arm-RB** (*i.e.*, **AGuIX Ln@HA-RB** and **AGuIX Ln@Ahx-RB**), **AGuIX Ln@Mal-K(RB)** and **AGuIX Ln@Mal-K(RB)DKPPR** is depicted in Scheme 3 (Ln = Tb or Gd).

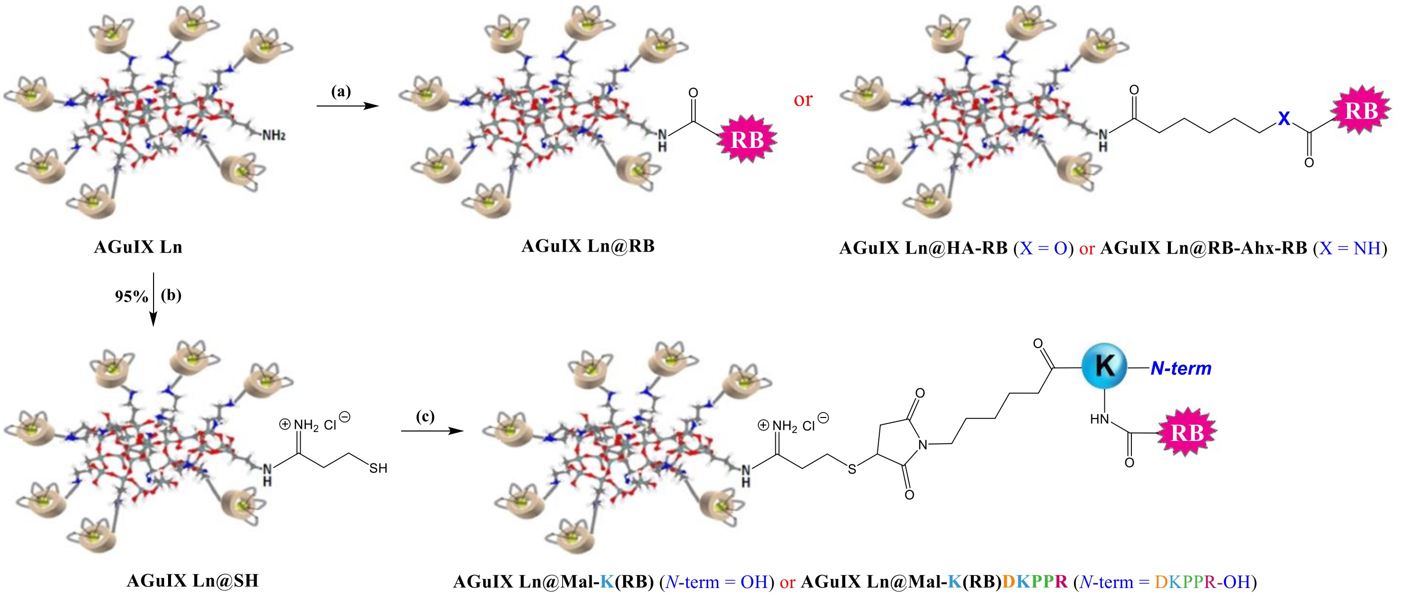

*Scheme 3: Synthesis of **AGuIX Ln@RB**, **AGuIX Ln@HA-RB**, **AGuIX Ln@Ahx-RB**, **AGuIX Ln@Mal-K(RB)** and **AGuIX Ln@Mal-K(RB)DKPPR** (Ln = Tb or Gd). Reagents and conditions: (a) i) H20, RT, 1 h, ii) NaOH (0.5 M), pH 8, iii) NHS-activated ester derivative of RB (**RB-NHS**, **RB-HA-NHS** or **RB-Ahx-NHS)** (0.06 eq), DMSO, RT, 3 h; (b) i) H20, RT, 1 h, ii) NaOH (0.5 M), pH 8, iii) Traut's reagent (1 eq), RT, 1 h; (c)) H20, NaOH (0.5 M), pH 8, ii) **Mal-K(RB)** or **Mal-K(RB)DKPPR**, DMSO, RT, 2.5 h.*

1.3.1. Synthesis of AGuIX Ln@RB, AGuIX Ln@HA-RB and AGuIX Ln@Ahx-RB

The **AGuIX Ln** NPs ([Ln^{3+}] = 50 mM) were synthesized as previously described [3] and provided by NH TherAGuIX.

AGuIX Ln NPs ([Ln^{3+}] = 50 mM) were dispersed in water and the solution was kept for 1 h stabilization at room temperature. The solution was adjusted to pH 8 using 0.5 M NaOH and then a solution of **RB-NHS**, or **RB-HA-NHS** or **RB-Ahx-NHS** (6 mol% in relation to Ln content) in DMSO (5% relative to the total volume of the solution) was added in five portions every 20 min (Scheme 3). After 3 h of stirring at room temperature, the NPs were diluted to reach [Ln^{3+}] = 10 mM and purified by tangential filtration over a 5 kDa cutoff Vivaspin membrane. Several tangential filtration cycles were carried out at 4500 rpm with an acceleration of 5 until a transparent filtrate was obtained. The filtrate was monitored by absorption spectroscopy. According to a calibration curve, the coupling of RB (or RB-HA or RB-Ahx) with the AGuIX Ln is considered complete when the filtrate shows no absorption of the precursors. Calibration curves indicate that the Ln:RB (or RB-HA or RB-Ahx) ratio is equal to 16:1 (corresponding to one RB derivative for one to two NPs). Six different NPs were thus obtained: **AGuIX Tb@RB**, **AGuIX Tb@HA-RB**, **AGuIX Tb@Ahx-RB**, **AGuIX Gd@RB**, **AGuIX Gd@HA-RB** and **AGuIX Gd@Ahx-RB**.

1.3.2. Synthesis of AGuIX Ln@K(RB) and AGuIX Ln@K(RB)DKPPR

First, **AGuIX Ln** NPs ([Ln^{3+}] = 50 mM) were thiolated using Traut's reagent (2-iminothiolane hydrochloride). **AGuIX Ln** NPs were dispsersed in water and the solution was kept for 1 h stabilization at room temperature. The solution was adjusted to pH 8 using 0.5 M NaOH and then a solution of Traut's reagent (100 mol% in relation to Ln content) in water was added dropwise (Scheme 3). After 1 h of stirring at 40°C, the desired **AGuIX Ln@SH** NPs were centrifugated at 4500 rpm with an acceleration of 5. The purification is completed after the disappearance of the specific UV-Visible absorption peak of the Traut's reagent at 248 nm. Thanks to a calibration curve, a Ln:SH ratio of 8:1 was estimated in **AGuIX Ln@SH** NPs.

Finally, **AGuIX Ln@SH** NPs were dispsersed in basic aqueous solution at pH 8 using 0.5 M NaOH and then a solution of **Mal-K(RB)** or **Mal-K(RB)DKPPR** in DMSO (5% relative to the total volume of the solution) was added in five portions during 30 min in the dark under stirring at room temperature (Scheme 3). After 2 h additional stirring, the solution was diluted in water to reach 4% final DMSO concentration. Purification was performed by tangential filtration over a 5 kDa cutoff Vivaspin membrane as previously described (see § **4.2.3.1.**). The solution of **AGuIX Ln@K(RB)** (or **AGuIX Ln@K(RB)DKPPR**) NPs was then concentrated to reach [Ln^{3+}] = 100 mM, adjusted to pH 7.2 and lyophylized for storing. Calibration curves indicate that one **Mal-K(RB)** (or **Mal-K(RB)DKPPR**) is present for 16 Ln^{3+} (corresponding to one to two NPs). Four different NPs were thus obtained: **AGuIX Tb@K(RB)**, **AGuIX Tb@K(RB)DKPPR**, **AGuIX Gd@K(RB)** and **AGuIX Gd@K(RB)DKPPR**.

2. Bimodal fitting of AGuIX-Tb NPs taylorgrams

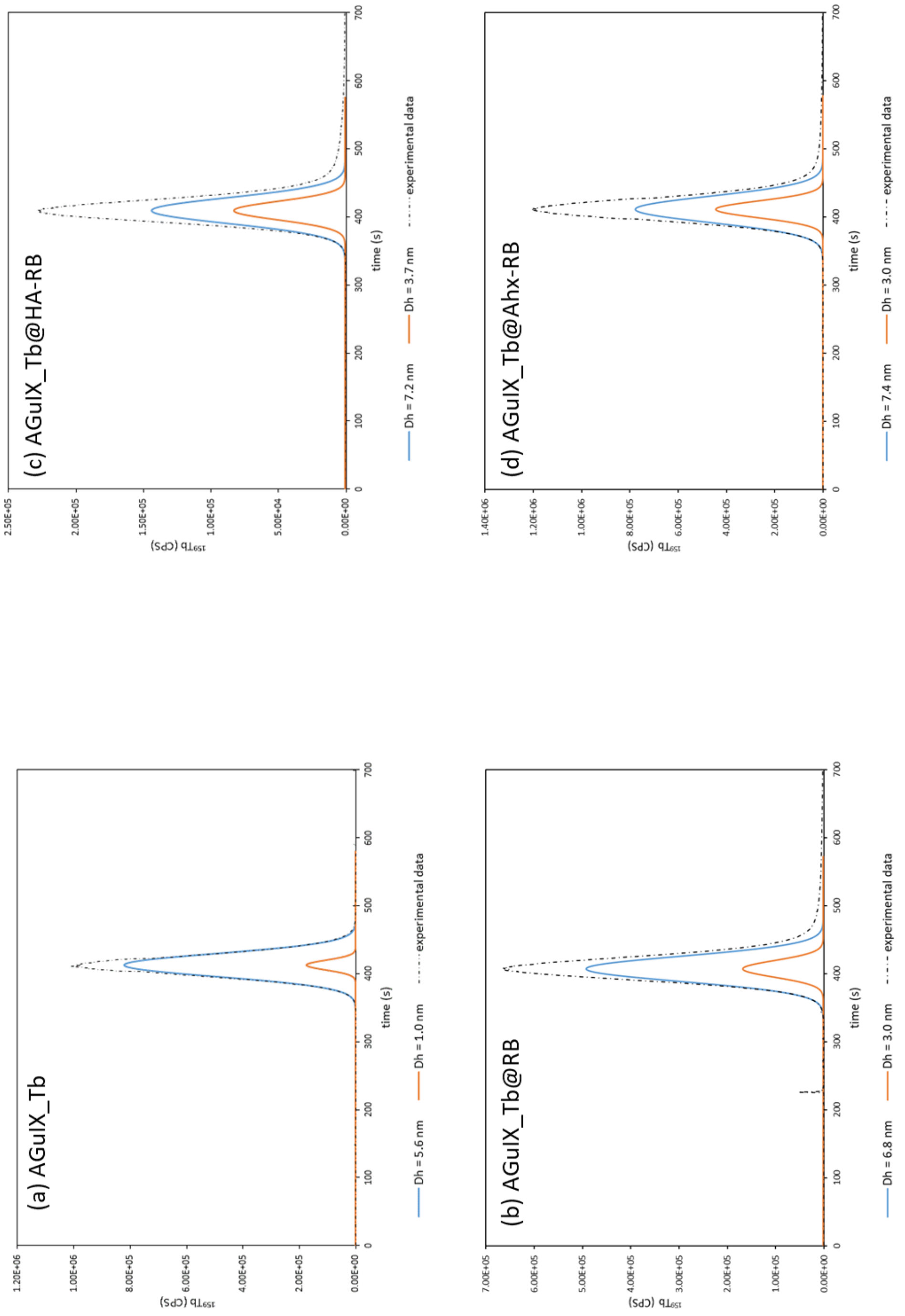

Figure 1: Bimodal fitting of Tb-AguIX NP staylorgrams. The signal (dashed black line) was fitted as the sum of two Gaussian curves (blue and orange lines)
